# Effectiveness of Benralizumab in Improving the Quality of Life of Severe Eosinophilic Asthmatic Patients: Our Real-Life Experience

**DOI:** 10.3389/fphar.2021.631660

**Published:** 2021-02-12

**Authors:** Giulia Scioscia, Giovanna Elisiana Carpagnano, Carla Maria Irene Quarato, Donato Lacedonia, Sonia Santamaria, Piera Soccio, Annarita Depalo, Paolo Fuso, Maria Pia Foschino Barbaro

**Affiliations:** ^1^ Department of Medical and Surgical Sciences, University of Foggia, Foggia, Italy; ^2^ Institute of Respiratory Diseases, Policlinico Universitario “Riuniti” di Foggia, Foggia, Italy; ^3^ Department of Basic Medical Sciences, Neuroscience and Sense Organs, Section of Respiratory Disease, University “Aldo Moro” of Bari, Bari, Italy

**Keywords:** severe asthma, eosinophilic asthma, benralizumab, quality of life, real-life

## Abstract

**Background:** Severe eosinophilic asthma decreases lung function and causes worsen symptoms, often forcing recurrent maintenance corticosteroid use. The aim of our real-life study was to evaluate the effectiveness of an add-on treatment with benralizumab in patients with severe eosinophilic asthma, paying particular attention to the impact on their quality of life (QoL).

**Materials and methods:** In this prospective study, 10 outpatients with severe eosinophilic asthma were added-on with benralizumab and followed-up in our severe asthma clinic after 12 and 24 weeks. At each patient visit, pre-bronchodilator FEV1 and inflammatory markers were recorded. Variations in asthma symptoms control and QoL perception was assessed by validated questionnaires.

**Results:** All the subjects experienced a marked reduction of nocturnal and diurnal symptoms over time and were able to stop using OCS, as documented by the improvement in Asthma control test (ACT) and Asthma Control Questionnaire score. Similarly, we recorded a statistically significant increase in patient’s QoL perception in EQ-VAS, EQ-5D-3L and Asthma Quality of Life Questionnaire (AQLQ) assessment (*p* < 0.05). Simultaneously we recorded a significant reduction in eosinophilic inflammation, an improvement in pre-bronchodilator FEV1. These results appear to be in line with those already obtained in the previous randomized controlled trials (RCTs).

**Conclusion:** Our 24-weeks real life experience supports the effectiveness of an add-on treatment with benralizumab in reducing eosinophilic inflammation and OCS-use, increasing lung function and improving control of nocturnal and diurnal symptoms, as well as restoring severe asthma patients to a better QoL.

## Highlights

1. Uncontrolled severe eosinophilic asthma deeply impacts on professional, social and family life of patients.
2. Benralizumab is a monoclonal antibody binding the alpha subunit of the IL-5 receptor that have yet demonstrated its effectiveness in multicentric phase III studies.
3. Real life studies better represent routine practice and can assess treatment patterns across a much broader range of outcomes.
4. A biological treatment with benralizumab in real-life seems to improve symptom control and the quality of life perceived by the patients over time, as highlighted by the improvement in the ACT, ACQ-6, AQLQ, EQ-VAS, EQ-5D-3L and Beck DI score.
5. Our real-life experience supports benralizumab effectiveness also in increasing pre-bronchodilator FEV1 and in reducing eosinophilic inflammation and OCS use.

## Introduction

A high level of eosinophils, present in at least 50% of patients with severe asthma, causes inflammation and hyperresponsiveness of the airways, worsening symptoms and decreasing lung function (Pavord, 2013). As a result, despite having the highest level of therapy required by the GINA guidelines (STEP 4–5) (“GINA Main Report, 2019”), patients with severe eosinophilic asthma present an increased risk of hospitalization, emergency room access and death (Pavord, 2013). Nevertheless professional, social and family life is deeply affected (Hossny et al., 2017). The two main aspects that potentially affect the quality of life (QoL) in patients with severe asthma are persistent diurnal and nocturnal symptoms and activity limitations (Stucky et al., 2015). In addition, the weight of asthma exacerbations affects the whole family, causing psychological tensions and possible financial problems. A good percentage of these patients rely on recurrent or maintenance oral corticosteroids (OCSs) use. Cumulative dosing from recurrent OCS use in the short term is also associated with adverse events (AEs) including osteoporosis, fractures, hyperglycemia, diabetes, cardiovascular disease, anxiety and immunosuppression (Price et al., 2018; Voorham et al., 2019). These adverse effects have a further negative impact on the QoL and can also worsen other comorbidities (Voorham et al., 2019).

Recent available OCS-sparing biological therapies for severe eosinophilic asthma are increasingly showing efficacy in terms of asthma control and enhanced quality of life (Chupp et al., 2017; O’quinn et al., 2019). Among them, benralizumab is a biological drug that is indicated as an additional maintenance treatment in adult patients with severe eosinophilic asthma not adequately controlled despite high-dose inhaled corticosteroids plus long-acting beta-agonists. It is a humanized monoclonal antibody (IgG1, kappa) which binds to the alpha subunit of the human receptor for interleukin 5 (IL-5Rα) with high affinity and specificity. As IL-5 is particularly important in the terminal differentiation, activation, recruitment and survival of committed eosinophil precursors, the IL-5 receptor is predominantly expressed on the surface of eosinophils. By blocking the signals of IL-5, Benralizumab targets key eosinophil activation and recruitment mechanisms. In addition, the absence of fucose in the Fc domain of benralizumab determines high affinity also for the FcɣRIII receptors present on immune effectors, including natural killer (NK) cells. This interaction causes apoptosis of eosinophils through enhanced cell-mediated antibody-dependent cytotoxicity (ADCC), which reduces eosinophilic inflammation (Pelaia et al., 2018).

The approval of benralizumab in the treatment of severe refractory eosinophilic asthma is based on the results of the WINDWARD program, which includes the two phase III studies on exacerbations, SIROCCO (Bleecker et al., 2016) and CALIMA (FitzGerald et al., 2016), and the phase III study on the decreasing in use of oral corticosteroids, ZONDA (Nair et al., 2017). In these studies, benralizumab has demonstrated its ability to induce eosinophil depletion in the blood, leading to a significant reduction in the annual exacerbation rate and improvement in pre-bronchodilator FEV_1_ and symptom control compared to placebo, especially in patients with more than 300 blood eosinophils/microliter in the pre-treatment period.

Outside of these randomized clinical trials (RCT), in recent times, real life studies on benralizumab effectiveness have been gaining increasing interest (Pelaia et al., 2019; Miralles López et al., 2020; Padilla-Galo et al., 2020; Pelaia et al., 2020). Indeed, compared with the idealized conditions of an RCT, real life studies better represent routine practice and can assess treatment patterns across a much broader range of outcomes.

On this background, the aim of this preliminary study was to present our real-life experience. As GINA Difficult to Treat and Severe asthma guidelines (GINA, 2019) recommend, we assessed response to benralizumab after 12 weeks and 24 weeks of treatment, paying particular attention to whether and how such add-on treatment may improve the quality of life (QoL) in patients with severe eosinophilic asthma.

## Materials and Methods

In this prospective observational real-life study, we enrolled 10 consecutive outpatients with diagnosis of severe eosinophilic asthma (Chung et al., 2014). Inclusion criteria were: age ≥18 years, smoking ≤10 P/Y, a not well controlled asthma despite taking daily maximal inhaled treatment plus another controller (according to STEP 4–5 of GINA guidelines) or OCS therapy for at least 6 months during the previous year, more than 300 eosinophils/mm^3^ of peripheral blood. Patients were excluded from the study if they were not completely adherent to their asthma maintenance prescribed therapy, showed an incorrect inhaler technique or if they had any other overlapping pulmonary condition (i.e., COPD, bronchiectasis, fibrosis) likely to interfere with results. This study was carried out according to the principles of the Declaration of Helsinki, was approved by the local ethics committee (institutional review board approval N°17/CE/2014), and all recruited patients gave their written informed consent.

Benralizumab was prescribed at the licensed dose of subcutaneous 30 mg according to the approved posology of a single subcutaneous injection, to be administered at intervals of 4 weeks for the first three times, and every 8 weeks thereafter. Each drug administration was performed in our outpatient clinic and patients were observed for at least an hour to record any immediate adverse reaction.

All the 10 patients included in the study, have been visited at baseline and after 12 and 24 weeks after starting treatment. At each patient visit the following parameters were recorded and comparatively evaluated: pre-bronchodilator forced expiratory volume in 1 s (FEV1), blood eosinophil count, number of exacerbations during the previous 24 weeks, fractional exhaled nitric oxide (FeNO), asthma control test (ACT) score, six items Asthma Control Questionnaire (ACQ-6) score, Asthma Quality of Life Questionnaire (AQLQ) score, EuroQol-Visual Analogue Scales (EQ-VAS) score, EuroQol-5Dimensions-3Levels (EQ-5D-3L) assessment and Beck Depression Inventory score (Beck DI). The doctor made his judgment on the clinical course of asthma by assigning a GETE (Global Evaluation on Treatment Effectiveness) score. Moreover, all enrolled patients were carefully checked in order to timely detect the eventual onset of side effects and adverse events.

### Asthma Control Test

ACT (Nathan et al., 2004) includes four questions on symptoms and the use of needed rescue drugs plus a self-assessment by the patient on the level of control. The score ranges from 5 to 25 (the higher the better). A score between 20 and 25 is expressions of well-controlled asthma, between 16 and 20 of poorly controlled asthma and between 5 and 15 of uncontrolled asthma. The minimal clinically important difference corresponds to three points.

### Asthma Control Questionnaire-6 Items

The ACQ-6 (Juniper et al., 1999) is calculated as the average of six items: it includes five questions about symptoms and one question about the use of rescue bronchodilator drugs. The score varies from 0 to 6 (the higher the worse). A score of 0.0–0.75 is indicative of a well-controlled asthma; a score of 0.75–1.5 represents a “gray” area; a score >1.5 indicates poorly controlled asthma. The clinically significant minimum difference is 0.5.

### Asthma Quality of Life Questionnaire

The AQLQ (Juniper et al., 1992) is calculated as the average of 32 items falling into four main domains: Symptoms (11 items), Activity Limitation (12 items, five of which are individualized), Emotional Function (5 items), and Environmental Exposure (4 items). Scores range is between 1 and 7, with higher scores indicating better quality of life. The clinically significant minimum difference is 0.5.

### EuroQol-Visual Analogue Scales and EuroQol-5Dimensions-3Levels

EQ-VAS (Brooks and De Charro, 1996) is a vertical visual analogue scale ranging from 0% (“worst possible”) to 100% (“best possible”) which asks patients to indicate their perceived overall health on.

The EQ-5D-3L (Brooks and De Charro, 1996) is a health status descriptive system that comprises the following five dimensions: mobility, self-care, daily activities, pain/discomfort and anxiety/depression. Each dimension has three levels: no problems, some problems, and severe problems. The patient is asked to indicate his/her health state by ticking the box next to the most appropriate statement in each of the five dimensions. This decision results in a 1-digit number that is finally combined into a 5-digit number describing the patient’s health state.

### Beck Depression Inventory

The Beck DI (Beck et al., 1961) is a 21-question multiple-choice self-report inventory for measuring the severity of depression. When the test is scored, a value of 0–3 is assigned for each answer and then the total score is compared to standard cut-off scores as follows: 0–9 indicates minimal depression; 10–18 indicates mild depression; 19–29 indicates moderate depression and 30–63 indicates severe depression.

### Global Evaluation on Treatment Effectiveness

GETE (Lloyd et al., 2007) is a simple asthma valuation tool in which the physician is asked to respond how effective the treatment has been in controlling the patient’s asthma. The five category scale responses include the following: Excellent = complete control of asthma; Good = marked improvement of asthma; Moderate = observable, but limited improvement in asthma; Poor = no appreciable change in asthma and Worsening = worsening of asthma.

### Statistical Analysis

Data at baseline were expressed as mean ± standard deviation (SD) for continuous variables and in terms of number and percent (n, %) for categorical variables. Statistical analysis to assess results obtained after therapy was performed by means a paired Student’s *t*-test for continuous variables and Chi-square test for categorical variables. A *p*-value lower than 0.05 was considered to be significant. Statistical analysis was performed using GraphPad Prism 5 (GraphPad Software Inc., La Jolla, CA, United States).

## Results

Demographic and clinical data at baseline of our patients are shown in Table 1.

**TABLE 1:** Characteristics of patients at baseline.

*Demographic Characteristic*	*Value*
Age, years (mean ± DS, min–max)	54 ± 8.8 (32–74)
Sex, female (n, %)	7 (70%)
Sex, male (n, %)	3 (30%)
BMI, kg/m^2^ (mean ± DS)	27.2 ± 5.1
Former smokers (n, %)	1 (10%)
Atopy (n, %)	4 (40%)
Positive skin prick test to a perennial aeroallergen (n, %)	4 (40%)
Positive skin prick test to a seasonal aeroallergen (n, %)	1 (10%)
OCS-dependent patients (n, %)	6 (60%)
Number of exacerbations/year pre-treatment (mean ± DS, min–max)	4.3 ± 0.96 (3–6)
*Comorbidities (n, %)*	
Nasal polyposis	7 (70%)
GER	6 (60%)
Bronchiectasis	1 (10%)
OSAS	1 (10%)
AERD	1 (10%)
*Questionaires (mean* ± *SD)*	
ACT	13.5 ± 1.5
ACQ-6	3.48 ± 0.65
AQLQ	3.65 ± 0.56
EQ-VAS	44.5 ± 7.7
BECK	14.6 ± 3.7
*Spirometry (mean* ± *SD)*	
Pre-bd FEV_1,_ L	1.50 ± 0.38
Pre-bd FEV_1,_ %	54.3 ± 11.18
FEV1/FVC, %	48.5 ± 9.6
Post-bd FEV1, L	1.86 ± 0.5
Post-bd FEV1, %	67.1 ± 15.1
*Inflammatory Markers (mean* ± *SD, min*–*max)*	
Total IgE count, IU/ml	395.8 ± 139.7 (38–658)
Blood eosinophils count, cell/mm^3^	793.8 ± 301.24 (440–2,300)
FeNO, ppb	46.79 ± 5.19 (35.41–57.4)
*Maintenance Therapy (n, %)*	
High-dose ICSs + LABAs	3 (30%)
High-dose ICSs + LABAs + LAMAs	7 (70%)

Abbreviations: OCS, Oral Corticosteroids; GER, Gastro-esophageal reflux; OSAS, Obstructive Sleep Apnea Syndrome; AERD, Aspirin-exacerbated respiratory disease; ACT, Asthma Control Test; ACQ-6, Asthma Control Questionnaire (6 items); AQLQ, Asthma Quality of Life Questionnaire; EQ-VAS,EuroQol-visual analogue scales; pre-bd FEV1, pre-bronchodilator forced expiratory volumein 1s; FENO_50_, Fraction of exhaled nitric oxide at 50 ml/s; ICSs inhaled corticosteroids; LABAs long-acting beta-adrenoceptor agonists; LAMAs long-acting muscarinic receptor antagonists.

**FIGURE 1:**
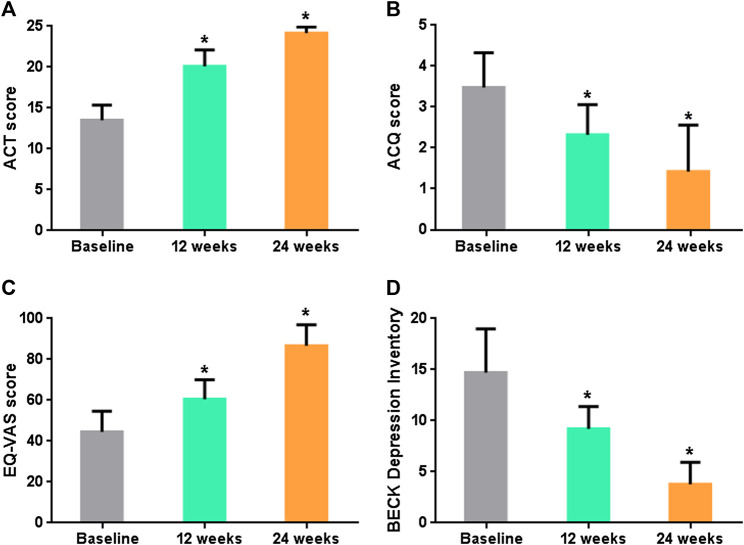
**(A)** Mean ACT score over time. **(B)** Mean ACQ score over time. **(C)** Mean EQ-VAS grade over time. **(D)** Mean Beck DI score over time. *p* < 0.05 is denoted by *.

ACT score enhanced from 13.5 ± 1.5 (baseline) to 20.3 ± 1.4 (12 weeks) (*p* < 0.0001) and to 24.2 ± 0.6 (24 weeks) (*p* < 0.0001), ACQ score reduced from 3.48 ± 0.65 (baseline) to 2.32 ± 0.57 (12 weeks) (*p* = 0.004) and to 1.42 ± 0.92 (24 weeks) (*p* < 0.0001), EQ-VAS increased from 44.5 ± 7.7 (baseline) to 60.5 ± 6.6 (12 weeks) (*p* = 0.002) and to 86.7 ± 7.2 (24 weeks) (*p* < 0.0001) and BECK DI reduced from 14.6 ± 3.68 (baseline) to 9.1 ± 1.64 (12 weeks) (*p* = 0.002) and to 3.7 ± 1.8 (24 weeks) (*p* < 0.0001) (Figures 1A–D).

**FIGURE 2:**
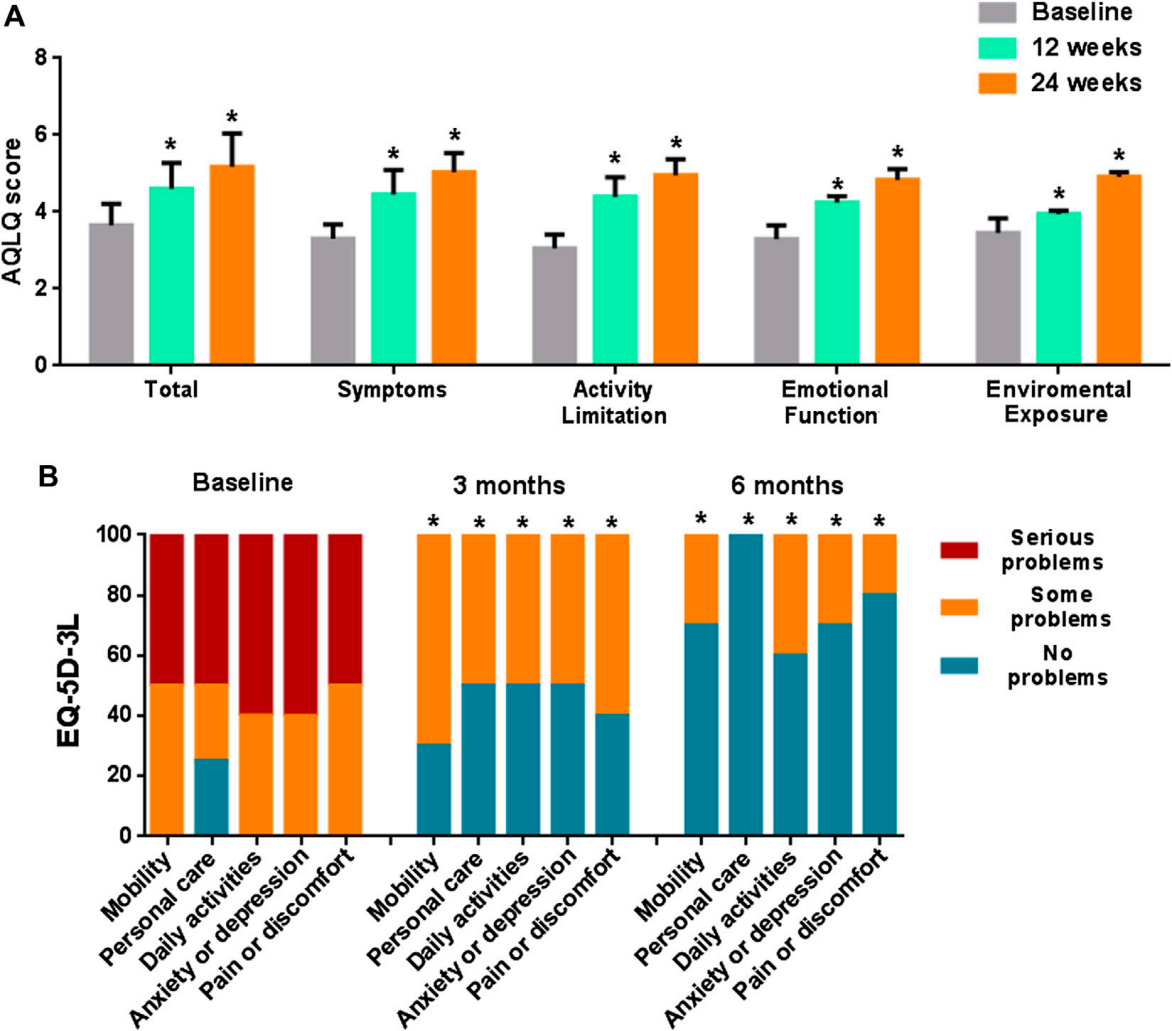
**(A)** Total and single domains AQLQ scores over time. **(B)** EQ-5D-3L assessment over time. Statistical significance compared with baseline (*p* < 0.05) is denoted by *.

AQLQ total score improved from 3.65 ± 0.56 (baseline) to 4.61 ± 0.67 (12 weeks) (*p* = 0.003) and to 5.17 ± 0.87 (24 weeks) (*p* = 0.0002), with a statistically significant increase in all domains over the time (Figure 2A). The percentage of patients complaining serious problems in all domains of the EQ-5D-3L at baseline significantly decreased both after 12 and after 24 weeks of treatment (*p* < 0.05) (Figure 2B).

No GETE assessment reported poor response or worsening of asthma for any patient at any time point in this study (Table 2).

**TABLE 2:** Global evaluation of treatment effectiveness.

*GETE, n (%)*	*12 weeks*	*24 weeks*
Excellent	2 (20%)	4 (40%)
Good	4 (40%)	6 (60%)
Moderate	4 (40%)	0 (0%)
Poor	0 (0%)	0 (0%)
Worsening	0 (0%)	0 (0%)

Abbreviations: GETE, Global Evaluation of Treatment Effectiveness; Excellent, total control of asthma; good, significant improvement of asthma; moderate, observable but limited improvement of asthma; poor, no noteworthy change of asthma; worsening, worsening of asthma.

**FIGURE 3:**
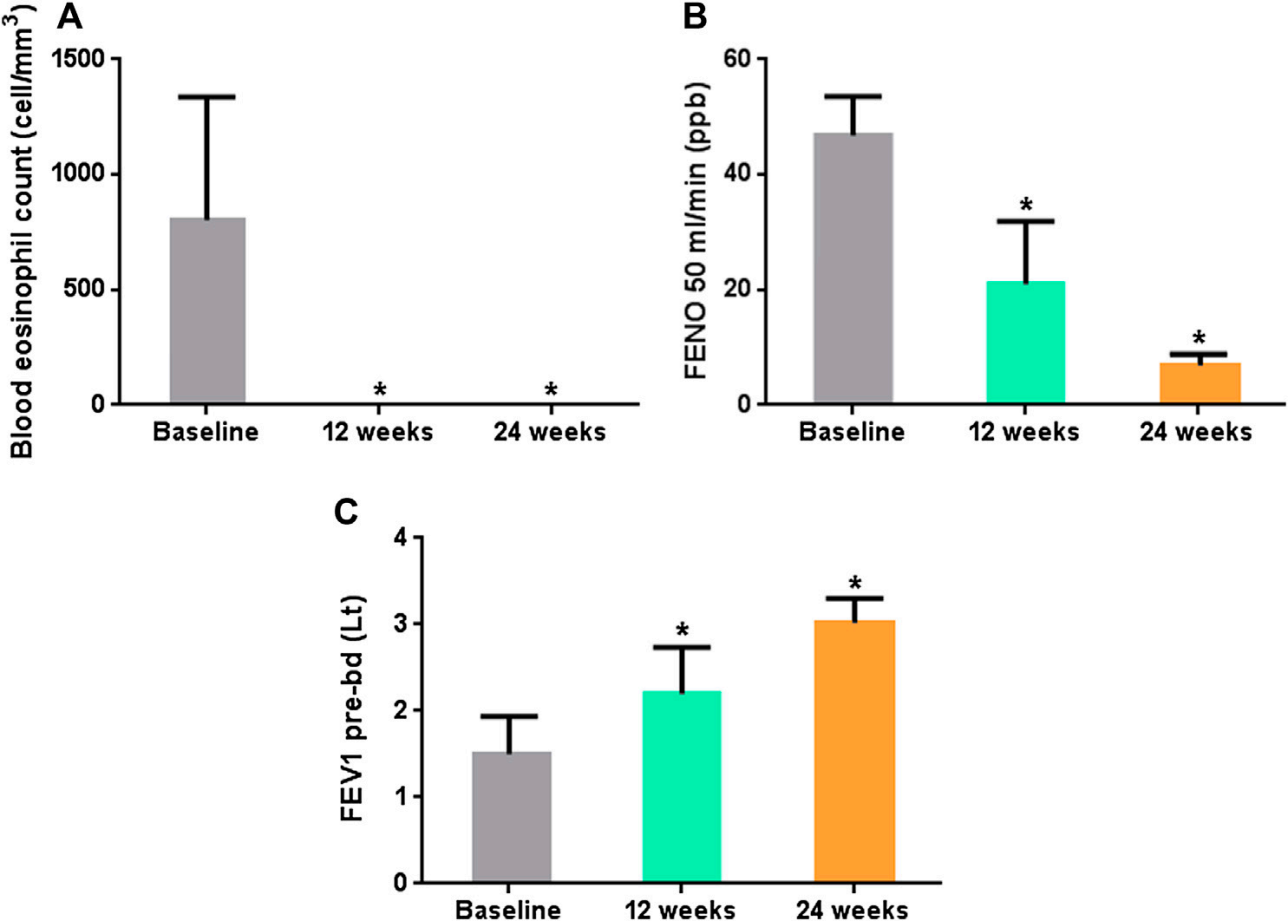
**(A)** Mean Blood eosinophil blood count over time. **(B)** Mean fractional exhaled nitric oxide at 50 ml/min (FeNO50) over time. **(C)** Mean prebronchodilator Forced Expiratory Flow in the first second (FEV1) in L/min over time. *p* < 0.05 is denoted by *.

The mean eosinophil blood count decreased from 793.8 ± 301.24 (baseline) to 0.25 ± 0.45 (*p* < 0.0001) and 0.0 ± 0.0 (*p* < 0.0001) after 12 and 24 weeks from the first subcutaneous injection of benralizumab, respectively. FeNO levels decreased from 46.79 ± 5.19 (baseline) to 20.68 ± 9.0 (12 weeks) (*p* < 0.0001) and to 6.69 ± 1.42 (24 weeks) (*p* < 0.0001) (Figures 3A, B). Such striking reduction in eosinophilic inflammation markers was associated to a significant increasing in the mean prebronchodilator FEV1 from 1.50 ± 0.38 L (baseline) to 2.21 ± 0.46 L (12 weeks) (*p* = 0.005) and to 3.02 ± 0.20 (24 weeks) (*p* < 0.0001) (Figure 1C). All the patients who were OCS-dependent at the baseline (6 patients) were able to discontinue such treatment after three months of treatment.

Clinical, functional and biological data over study time are summarized in Table 3.

**TABLE 3:** Clinical, functional and biological data over study time. All values are express as median ±SD (min–max).

	Baseline	T1 (12 weeks)	T2 (24 weeks)	*p* Value (baseline vs. T1)	*p* Value (baseline vs. T2)
ACT score	13.5 ± 1.5	20.3 ± 1.4	24.2 ± 0.6	<0.0001	<0.0001
ACQ score	3.48 ± 0.65	2.32 ± 0.57	1.42 ± 0.92	0.0005	<0.0001
AQLQ score:					
Total	3.65 ± 0.56	4.61 ± 0.67	5.17 ± 0.50	0.003	0.0002
Symptoms	3.31 ± 0.36	4.45 ± 0.64	5.03 ± 0.50	0.0001	<0.0001
Activity limitation	3.06 ± 0.35	4.39 ± 0.52	4.95 ± 0.42	<0.0001	<0.0001
Emotional function	3.30 ± 0.36	4.24 ± 0.17	4.83 ± 0.28	<0.0001	<0.0001
Enviromental exposure	3.45 ± 0.38	3.94 ± 0.09	4.92 ± 0.11	0.0009	<0.0001
EQ-VAS	44.5 ± 7.7	60.5 ± 6.6	86.7 ± 7.2	<0.0001	<0.0001
BECK DI	14.6 ± 3.68	9.1 ± 1.64	3.7 ± 1.8	0.002	<0.0001
FEV_1_ (pre-bd L) mean ± SD	1.50 ± 0.38	2.21 ± 0.46	3.02 ± 0.20	0.005	<0.0001
FEV_1_ (pre-bd %)	54.3 ± 11.2	78.3 ± 10.9	94.7 ± 11.5	0.0001	<0.0001
Blood eosinophils; cells/mm^3^	793.8 ± 301.24	0.25 ± 0.45	0.0 ± 0.0	<0.0001	<0.0001
Blood eosinophils; %	8.96 ± 4.86	0.55 ± 0.86	0.0 ± 0.0	<0.0001	<0.0001
FENO_50_; ppb	46.79 ± 5.19	20.68 ± 9.0	6.69 ± 1.42	<0.0001	<0.0001

Abbreviations: ACT, Asthma Control Test; ACQ-6, Asthma Control Questionnaire (6 items); AQLQ, Asthma Quality of Life Questionnaire; EQ-VAS,EuroQol-visual analogue scales; pre-bd FEV1, pre-bronchodilator forced expiratory volumein 1s; FENO_50_, Fraction of exhaled nitric oxide at 50 ml/s.

Treatment with benralizumab was safe and well tolerated, with only one patient complaining a self-resolved episode of headache the evening after the first dose injection. After 24 weeks of treatment, only three patients reported mild exacerbations (with a maximum of two in one of them). However, these episodes of exacerbation not required OCS-use and not penalized lung function improvement, resulting from an infectious nature and resolving after appropriate antibiotic therapy.

## Discussion

The most relevant aspect of this real-life study regarded asthma control and QoL improvement during an add-on treatment with benralizumab. Severe asthma, especially if poorly controlled, can be very limiting for the normal performance of daily activities due to limited social and physical activity and poor sleep quality with consequent great number of work absences, isolation and reduction in the QoL. In addition, the weight of asthma exacerbations affects the whole family, causing psychological tensions and possible financial problems.

In our real-life study all the 10 subjected enrolled experienced with Benralizumab a marked reduction of daily symptoms, nocturnal awakenings and rescue medicine need after 12 weeks of treatment, as documented by the improvement in ACT and ACQ score. The very rapid achievement of daily and nocturnal symptom control experienced by our patients is consistent with a recent analysis of pooled results from the SIROCCO and CALIMA phase III studies (O’quinn et al., 2019). In particular, the ACT score reached the cut-off of 20 points after 12 weeks of treatment and continued to increase at 24 weeks. Nevertheless, GETE physician’s assessment reported a moderate to excellent control of asthma for any patient at any time point of this study. Global evaluation of treatment effectiveness (GETE) is a validated tool in patients with moderate-severe allergic asthma, that has been used to evaluate the clinical response to omalizumab (Bousquet et al., 2014). Currently, in Italy, the clinician is required to express his opinion on asthma control at each renewal of the treatment plan for omalizumab, but this evaluation tool can be used for all biologics (Lloyd et al., 2007). The combined use of such assessment questionnaires gave us the possibility to obtain a feedback on treatment effectiveness by both the patient’s and the physician’s point of view and may be useful to employ in clinical practice to confirm clinical outcomes.

Similarly, we recorded a statistically significant improvement in patient’s perception QoL, as highlighted by the increasing in EQ-VAS, EQ-5D-3L and AQLQ score. Regarding AQLQ, such improvement covered all of the four health domains investigated among which symptoms perception, activity limitation, emotional function and environmental stimuli with a statistically significant improvement after 12 and 24 weeks with respect to baseline.

Sandy Khurana et al. (Khurana et al., 2019) have previously highlighted that worse respiratory symptoms-related QoL may be associated with an increased severity of depression in asthma patients. On the other hand, the increased feeling of anxiety and depression associated with severe asthma may also further lower QoL (Hossny et al., 2017). In our real-life experience, we recorded a progressive mood improvement over time with benralizumab. Also this factor may have contributed to the improvement in symptoms’ perception and QoL. Nevertheless, all the six patients who were OCS-dependent at the baseline were able to discontinue such treatment after three months of treatment yet. Indeed, the excellent improvement in symptom control made it possible for our patients a faster OCS withdrawal than that one reported by the phase III ZONDA trial (Nair et al., 2017). In the short follow-up period of 24 weeks covered by this study, the main advantage after tapering off the medication for all the patients was to have faced the satisfaction of weight loss. This is another important aspect to considerate in the improvement in the activity limitation’s perception and in the emotional sphere assessed by both the EQ-5D-3L and the AQLQ score. Probably a wider follow-up could highlight further long-term benefits that it would be very interesting to investigate in order to strengthen the evidence of potential benefits deriving by a biologic therapy vs. OCS-use.

The best known characteristic of benralizumab is its reported capacity to induce a fast depletion of eosinophils in blood that has to be related to the unique design and mechanism of action of this monoclonal antibody (Sridhar et al., 2019). Interestingly, the remarkable fall of blood eosinophils in our patients was associated with a parallel prompt improvement in airflow obstruction and therefore it may be supposed that such important effect of benralizumab on lung function is to relate to a quick resolution of bronchial eosinophilic inflammation. This finding support the excellent efficacy of benralizumab in improving lung function in patients with severe eosinophilic asthma as reported by randomized controlled trials (Chia et al., 2019). The average FEV1 improvements recorded in our study was largely greater than that observed during pre-marketing randomized controlled trials. However, in a pooled analysis of results from the SIROCCO (Bleecker et al., 2016) and CALIMA (FitzGerald et al., 2016) phase III studies benralizumab appeared more effective in improving lung function in patients with an eosinophil count ≥300 cells/μl (Bleecker et al., 2018). In our population the median eosinophil count at baseline was 794 cells/mm^3^, and this could have enhanced the beneficial effects of such target treatment. Overall, these results on pulmonary function represent a further objective confirmation for the improvement experienced by patients in terms of asthma control and Qol improvement.

In literature it is also reported an almost complete depletion of eosinophils in sputum and tissues (90 and 96%, respectively) with benralizumab (Ghazi et al., 2012). Unfortunately, not all the patients were able to perform the induced sputum examination during the different control visits and it was not possible to analyze the sputum eosinophilia variation due to lack of data. However other studies like this would still be desirable, considering that increased sputum eosinophilia might be a predictor of exacerbations more than blood eosinophil count (Ghazi et al., 2012). Despite this, patients in our study showed an important reduction in FeNO levels, that is another quite accurate predictor of eosinophilic airway inflammation (Gao and Wu, 2018), thus confirming this suggestion.

Globally treatment with benralizumab appeared safe and well tolerated in our study. After 24 weeks of treatment, three patients reported mild exacerbations (with a maximum of two in one patients), not requiring OCS-use and not penalizing lung function improvement over time. In most cases these were infectious exacerbations, which arose in the winter months and resolved after appropriate antibiotic therapy. Despite the scarce clinical importance of such episodes of exacerbation in our study, it is interesting to remember that Poznanski et al. (Poznanski et al., 2020) in a recent real life experience recorded a large number of non-eosinophilic exacerbation of infectious origin in patients treated with benralizumab. The authors explained this outcome with sputum eosinophils decrease and NK-cells disfunction. This may be another aspect that deserves of further investigation in other studies in real life.

The main strength of our study is to have focused on the benefit of a biological treatment for severe asthma in terms of important clinical aspects, such as symptom control and improvement on quality of life, that are the most perceived results by the patient. Taking into account that the observation period is relatively short, it is possible to hypothesize that the beneficial effects of benralizumab may already be observable after 12 weeks. However, even if this could be considered a strength, it could also be considered as a limitation, since it is not possible to establish whether such benefits persist over longer observation times. Another limitation of our study has to do with the low number of patients enrolled and in the lack of inclusion of all possible comorbidities of the study population (limiting it to rhinosinusitis with polyposis). Anyhow, this limit is in line with the fact that this was the representation of a real-life experience in a dedicated single-center clinic, which makes it more difficult to collect a large number of patients. Furthermore, a placebo effect could not be assessed due to the lack of a placebo control group. Also this limitation can be justified considering that placebo use is unethical in a real-life setting when a proven effective therapy is available. Despite this, it was encouraging to find that also other real-life studies on benralizumab have recorded a similar greater improvement in clinical parameters of their severe asthma patients, also in a shorter follow-up period, compared to the pivotal studies (Pelaia et al., 2019; Miralles López et al., 2020; Padilla-Galo et al., 2020; Pelaia et al., 2020). Some authors have explained such discrepancy with a higher compliance commonly manifested by outpatients in taking their maintenance therapy and in attending routine medical controls, rather than those being enrolled in randomized clinical trials (Pelaia et al., 2020). In addition, our study may have been biases by the fact that the majority of our patients (70%) had nasal polyposis, which is the most common comorbidity associated with severe eosinophilic asthma. Data in literature have shown that the presence of nasal polyps represents a predictive factor of a successful therapeutic response to an add-on treatment with benralizumab (Bleecker et al., 2018. Indeed, chronic rhinosinusitis with nasal polyps showed some characteristics similar to eosinophilic asthma, such as eosinophilic pattern of inflammation and the possible association with atopy or aspirin sensitivity. Therefore, improvement in asthma control in patients with severe eosinophilic asthma and associated nasal polyps could be related to the simultaneous improvement of this comorbidity. Anyhow, to have evaluated the benefit that benralizumab in this group of patients, can be considered also a strength of our study. Indeed, it gave us a further evidence of advantage on the use of this biological drug in the presence of nasal polyps. This may help to orient the choice of the clinician toward the most appropriate biological drug in patients with this comorbidity, in order to develop a more precise and “personalized” treatment for severe asthma.

## Conclusion

In conclusion, our real-life experience supports the effectiveness of benralizumab as an add-on biological treatment in improving nocturnal and diurnal symptom control and mood, as well as restoring patients with severe eosinophilic asthma to an acceptable QoL, as highlighted by the increasing in the ACT, ACQ-6, AQLQ, EQ-VAS, EQ-5D-3L and Beck DI score. It also resulted in an increased lung function and a reduction in eosinophilic inflammation and OCS-use, already only after 12 weeks. These results appear to be in line with those already obtained in the previous randomized controlled trials (RCTs) and real life studies. Benralizumab would represent an optimal choice for improving the clinical treatment and management of patients with severe eosinophilic asthma, especially in those who shows a comorbidity condition of nasal polyps.

## Data Availability

The raw data supporting the conclusions of this article will be made available by the authors, without undue reservation.

## Abbreviations:

ACT, Asthma control test; ACQ-6, Asthma control questionnaire-6 items; AEs, adverse events; Beck DI, Beck Depression Inventory; EQ-5D-3L, EuroQol-5 Dimensions-3 Levels; EQ-VAS, EuroQol-visual analogue scales; FENO50, Fraction of exhaled nitric oxide at 50 ml/s; FEV, forced expiratory volume in 1s; FVC, forced vital capacity; GETE, Global Evaluation on Treatment Effectiveness; ICS, inhaled corticosteroids; LABAs, long-acting beta-adrenoceptor agonists; LAMAs, long-acting muscarinic receptor antagonists; OCS, oral corticosteroid; QoL, quality of life.
